# Neuroprotective effects and mechanism of cognitive-enhancing choline analogs JWB 1-84-1 and JAY 2-22-33 in neuronal culture and Caenorhabditis elegans

**DOI:** 10.1186/1750-1326-5-59

**Published:** 2010-12-16

**Authors:** Roongpetch Keowkase, Marwa Aboukhatwa, Bao-Ling Adam, J Warren Beach, Alvin V Terry, Jerry J Buccafussco, Yuan Luo

**Affiliations:** 1 Department of Pharmaceutical Sciences, School of Pharmacy, University of Maryland, Baltimore, MD, USA; 2 Department of Pharmacology and Toxicology, Medical College of Georgia, Augusta GA 30912, USA; 3 Department of Pharmaceutical and Biomedical Sciences, college of Pharmacy, University of Georgia, Athens, GA 30602, USA; 4 Center for Integrative Medicine, School of Medicine University of Maryland, Baltimore, MD, USA; 5 Faculty of Pharmacy, Srinakharinwirot University, 63 Rangsit-Nakornayok Rd., A. Ongkarak, Nakornayok, 26120, Thailand

## Abstract

**Background:**

Our previous work indicated that novel analogs of choline have cytoprotective effects *in vitro *that might be useful in neurodegenerative conditions such as Alzheimer's disease (AD). Furthermore, two lead compounds (JWB1-84-1 and JAY2-22-33) from a library of more than 50 improved cognitive performances in a transgenic mouse model of AD. The purpose of these experiments was to more specifically investigate the neuroprotective capabilities of these lead compounds both *in vitro *and *in vivo*.

**Results:**

We used N2a cells which express a Swedish mutation in the amyloid precursor protein and presenilin 1 genes to investigate the effect of JWB1-84-1 and JAY2-22-33 on β-amyloid (Aβ) levels and found that both compounds significantly reduced Aβ levels. JWB1-84-1 and JAY2-22-33 also protected rat primary cortical neurons from Aβ toxicity. Subsequently, we utilized the nematode *Caenorhabditis elegans *(*C. elegans*) as an *in vivo *model organism to identify potential molecular targets of these compounds. In the *C. elegans *model of Aβ toxicity, human Aβ is expressed intracellularly in the body wall muscle. The expression and subsequent aggregation of Aβ in the muscle leads to progressive paralysis.

**Conclusion:**

We found that JAY2-22-33 (but not JWB1-84-1) significantly reduced Aβ toxicity by delaying paralysis and this protective effect required both the insulin signaling pathway and nicotinic acetylcholine receptors (nAChRs).

## Background

Alzheimer's disease (AD) is a progressive neurodegenerative disease that is believed to be caused by the abnormal aggregation of harmful proteins including β-amyloid (Aβ) peptide and microtubule-associated protein tau [1,2]. The treatment of AD is currently limited to the symptomatic approaches with 2 classes of FDA approved drugs, acetylcholinesterase inhibitors (AChEIs) and NMDA receptor antagonist [3]. The Alzheimer's Association estimates that by the year 2050, without better ways to prevent the disease, the number of AD patients will be increased to between 11 and 16 million in the United States and more than 100 million worldwide [4]. This leads to the need for the development of effective compounds that can provide disease-modifying property.

Nicotine has been shown to improve performance on attention and memory tasks both in humans and animal subjects [5]. In addition, many studies have indicated that nicotine could have a potential therapeutic benefit in treating AD since it has been shown to reduce Aβ levels in both rat and mouse models of AD [6,7]. The neuroprotective effects of nicotine are thought to be mediated via effects at α7 nicotinic acetylcholine receptor (nAChR) [6]. This receptor is involved in learning and memory and has been implicated in the pathophysiology of AD. It has been reported that the brain of AD patients and animal models of AD exhibit marked deceases in nAChRs especially α7- and α4β2-nAChRs [8] and the loss of these receptors is correlated with learning and memory deficits [9]. Therefore, nAChRs should be one of the therapeutic targets for the treatment of AD. This leads to a reasonable rationale for designing drugs with activity at nAChRs especially the α7 subtype.

**Figure 1:**
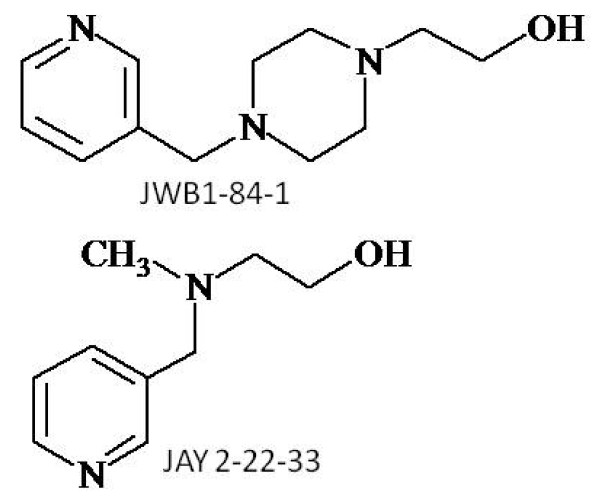
**The chemical structure of JWB1-84-1 and JAY2-22-33**.

Choline, a precursor of acetylcholine (ACh) and a product of acetylcholine hydrolysis by acetylcholinesterase (AChE), is a selective agonist of α7 nAChR [10,11]. Choline, like nicotine, exhibited a protective effect against cytotoxicity induced by growth factor deprivation in differentiated PC-12 cells [12]. So far, we have developed over 50 choline analogs with similar or greater potency than nicotine. These compounds produced cytoprotective effect with differences in potency and efficacy [13]. Among the series of synthetic choline analogs, two lead compounds, JWB1-84-1 and JAY2-22-33 (Figure 1) were also studied for other pharmacological properties. JWB1-84-1 improved cognitive performance in a transgenic mouse model of AD and significantly reversed distractor-impaired accuracies in an attention deficit model in young macaques [14]. JAY2-22-33 exhibited similar properties in this model (unpublished data).

In this paper, we studied the effects of JWB1-84-1 and JAY2-22-33 in both *in vitro *and *in vivo *models of AD. We used N2a cell which expresses a Swedish mutation in amyloid precursor protein and presenilin 1 genes to study the effect of compounds on Aβ levels and we used rat primary cortical neuron to study the neuroprotective effect of compounds on Aβ toxicity and we used the nematode *Caenorhabditis elegans *(*C. elegans*) as a model organism to identify the potential molecular targets of these compounds. *C. elegans *is a useful model to study the molecular mechanisms of drug action and has been used as a model for various age-associated neurodegenerative diseases [15], including Alzheimer's disease [16], Parkinson's disease [17] and Huntington's disease [18]. The transgenic *C. elegans *model of Aβ toxicity has been developed by expressing human Aβ in the muscle. The expression and subsequent aggregation of Aβ in the muscle lead to progressive paralysis [19]. The Aβ being expressed in this transgenic *C. elegans *is not full-length 1-42 but rather a 3-42 truncation product. *In vitro *analysis demonstrates that Aβ_3-42 _self-aggregates like Aβ _1-42_, but more rapidly, and forms fibrillar structures [20]. By using this model, we could examine the protective effect of JWB1-84-1 and JAY2-22-33 via the potential reduction of Aβ toxicity. Then, we could identify the molecular targets of these compounds by taking advantage of genetic amenability in this model.

In *C. elegans*, the insulin/IGF-1 signaling pathway controls many biological processes such as life span, metabolism and stress response. This pathway is comprised of many proteins including insulin/IGF-1 receptor (DAF-2), PI3-kinase (AGE-1) and FOXO transcription factor (DAF-16) and heat shock factor (HSF)-1. Recently, it was reported that modulation of the insulin/IGF-1 signaling pathway delayed the onset of Aβ toxicity in *C. elegans *expressing human Aβ [21,22]. To alleviate proteotoxicity, the insulin signaling requires HSF-1 to modulate Aβ disaggregation process, while DAF-16 regulates the less toxic high molecular aggregation process. In this study, we sought to determine whether JWB1-84-1 and JAY2-22-33 protects against Aβ toxicity by regulating these target genes, and whether the observed protective effect (if present) would be absent in nAChR mutants. Such information would begin to identify potential multitarget mechanisms that mediate the neuroprotective effect of choline analogs.

## Results and Discussion

### Nicotine and choline analogs protect primary cortical neuron from Aβ toxicity

**Figure 2:**
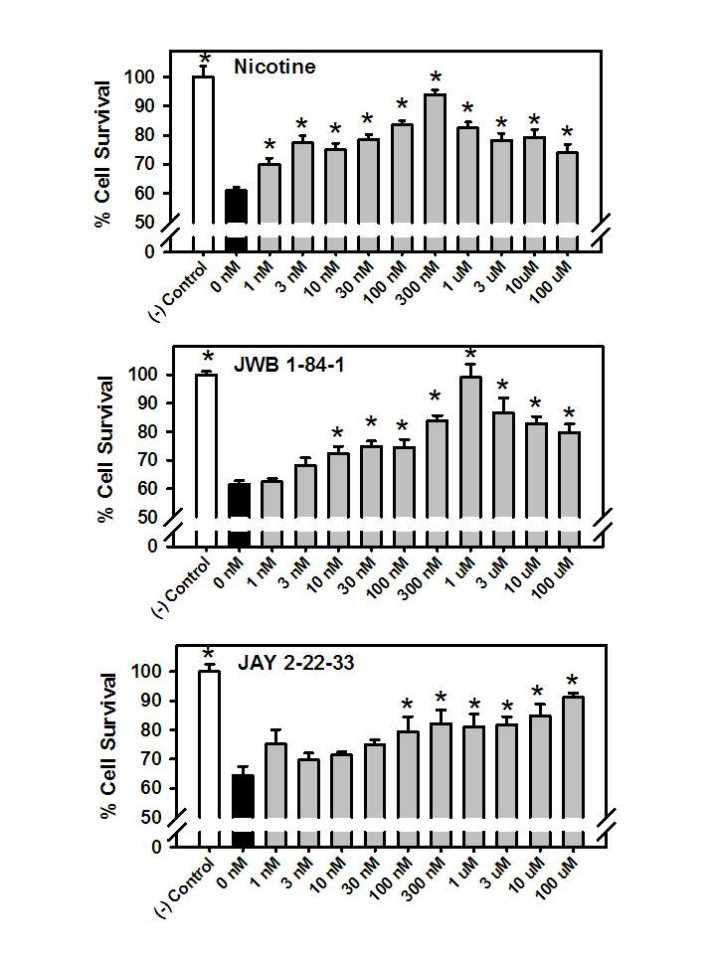
**Neuroprotective effects of nicotine, JWB 1-84-1, and JAY 2-22-33 against the Aβ_1-42 _peptide as determined in a cell viability assay in rat cortical neuron primary culture**. Pretreatment of the cultures with various concentrations of nicotine, JWB 1-84-1, and JAY 2-22-33 for 24 hours was followed by exposure to the Aβ_1-42 _peptide (100 nM) for another 24 hours. Cell viability for each treatment was calculated as percentage survival rate and compared to a negative control (i.e., cultures without the Aβ_1-42 _peptide, nicotine or choline analog). Each bar represents the mean ± S.E.M derived from at least 7 replicates per concentration and each experiment was repeated at least 2-3 times per drug. *p < 0.05 compared to wells with the Aβ_1-42 _peptide, but no nicotine or choline analog.

The results of experiments designed to assess the potential neuroprotective effects of nicotine and the choline analogs against the compromised neuronal viability induced by the Aβ_1-42 _peptide are illustrated in Figure 2. As illustrated, 24 hr incubation with the Aβ_1-42 _peptide (100 nM) decreased cell survival by about 40% in each series of experiments. Nicotine and each of the choline analogs significantly protected against Aβ-induced neurotoxicity. In fact, all of the concentrations of nicotine evaluated (1.0 nM to 100 μM) offered some degree of protection (*p *< 0.05). Likewise, in the case of JWB1-84-1, all of the concentrations evaluated above 3.0 nM levels offered protection (*p *< 0.05). For both nicotine and JWB1-84-1 there was an inverted U concentration-effect relationship with maximum levels of protection observed at 300 nM and 1.0 μM, respectively. In the case of JAY2-22-23, all of the concentrations higher than 30 nM produced statistically significant neuroprotective effects (*p *< 0.05). JAY2-22-23 did not produce an inverted U concentration-effect relationship and the highest concentration evaluated (100 μM) produced the highest degree of protection of approximately 92% cell survival.

### JWB1-84-1 and JAY2-22-33 reduced Aβ in medium of Aβ-expressing neuroblastoma cells

**Figure 3:**
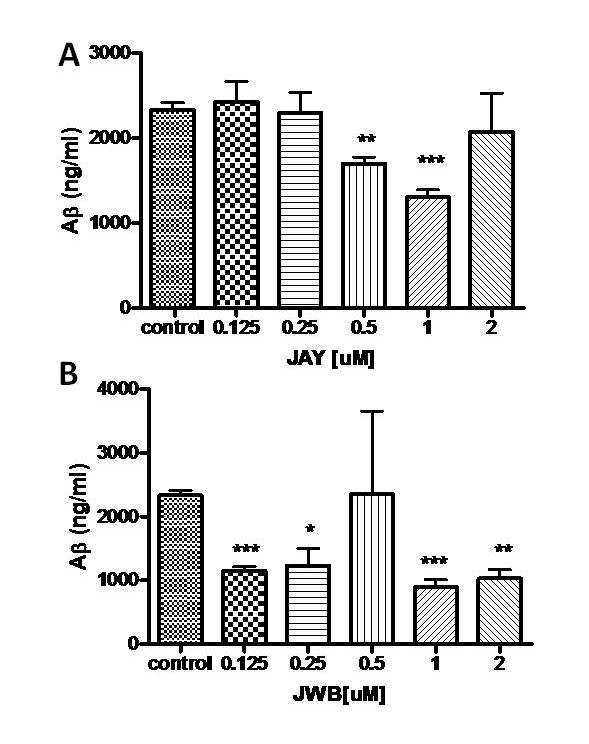
**Effect of JAY2-22-33 and JWB1-84-1 on Aβ levels using ELISA**. JAY2-22-33 and JWB1-84-1 treatment to N2a neuroblastoma cells significantly reduced secreted amount of Aβ monomers using ELISA (*p *< 0.05) based on one way ANOVA analysis. *Posthoc *dunnette test showed that the JAY2-22-33 treatment for 24 hours significantly reduced the levels of secreted Aβ monomers at 0.5 μM (*p *< 0.01) and 1 μM (*p *< 0.001). JWB treatment for 24 hours significantly reduced the amount of secreted Aβ monomers at 0.125 μM (*p *< 0.001), 0.25 μM (*p *< 0.05), 1 μM (*p *< 0.001) and 2 μM (*p *< 0.01). Data were expressed as mean± SEM (N = 3).

To test the hypothesis that JAY2-22-33 and JWB1-84-1 may have a possible role in modulating the amyloid peptide species as a mechanism underlying their beneficial effects in improved cognitive performance in transgenic mice model of Alzheimer's disease [14], N2a neuroblastoma cells which express the Aβ transgene after the addition of sodium butyrate were used. After induction by sodium butyrate, these mutant neuroblastoma cells were able to process the amyloid precursor protein to produce Aβ. N2a cells were treated with increasing concentration of either JAY2-22-33 or JWB1-84-1. Then the effect of JAY2-22-33 and JWB1-84-1 Aβ levels were determined by using ELISA. We found that JAY2-22-33 at concentration 0.5 and 1 μM and JWB1-84-1 at concentration 0.125, 0.25, 1 and 2 μM significantly reduced the level of Aβ in the medium (Figure 3A and 3B).

### Nicotine and JAY2-22-33, but not JWB1-84-1, delayed Aβ-induced paralysis in *C. elegans *strain CL2006

**Figure 4:**
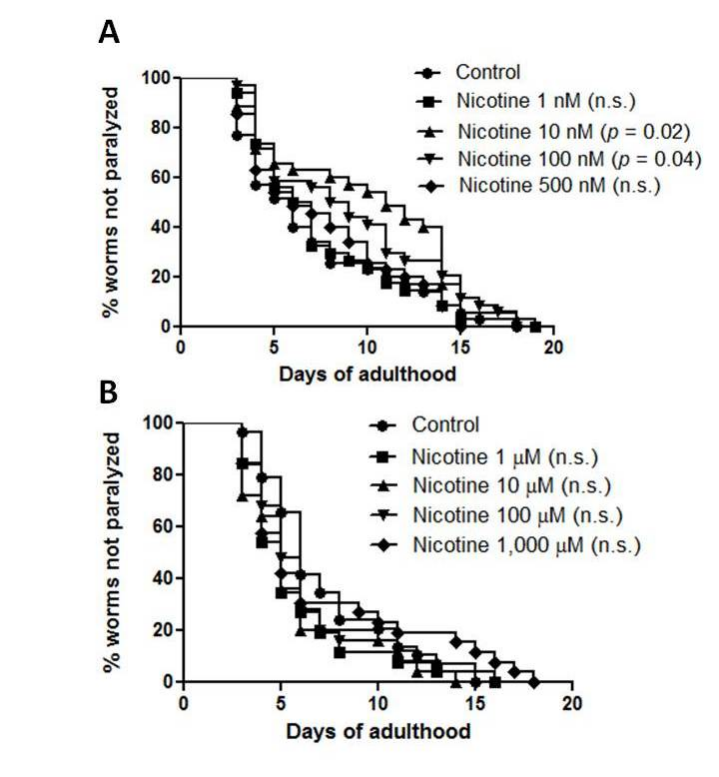
**Aβ-induced paralysis in *C. elegans *strain CL2006 fed with nicotine**. (A) Effect of nicotine at concentration 1, 10, 100 and 500 nm on Aβ-induced paralysis. (B) Effect of nicotine at concentration 1, 10, 100 and 1,000 μM on Aβ-induced paralysis. Synchronized eggs of CL2006 *C. elegans *were maintained at 20°C, on the 35 × 10 mm culture plates (~35 eggs/plate) containing vehicle (control) or nicotine. The treatment was given to the worm from egg stage onward until the worm completely paralyzed (35 worms in each experiment, 3 independent experiments). n.s. = not significant

According to the amyloid hypothesis, AD is thought to be caused by the production and deposition of neurotoxic Aβ-peptide in the brain [2]. The deposition of Aβ in the brain leads to many consequences such as the formation of neurofibrillary tangles, oxidative stress, glutamatergic excitotoxicity, inflammation, neuronal cell death and eventually the clinical symptoms of AD [3,23]. In transgenic *C. elegans *model of AD, human Aβ42 protein has been expressed intracellularly in the body wall muscle and the expression and subsequent aggregation of Aβ in the muscle lead to progressive paralysis. To investigate the protective effect of nicotine, the worm strain CL2006 which produces Aβ constitutively in the muscle was used. The worms were treated with nicotine at concentration ranging from 1 nM to 1 mM. We found that nicotine at concentration 10 and 100 nM significantly delayed Aβ-induced paralysis in this transgenic worm (*p*= 0.02 and 0.04, respectively, Figure 4A and 4B).

**Figure 5:**
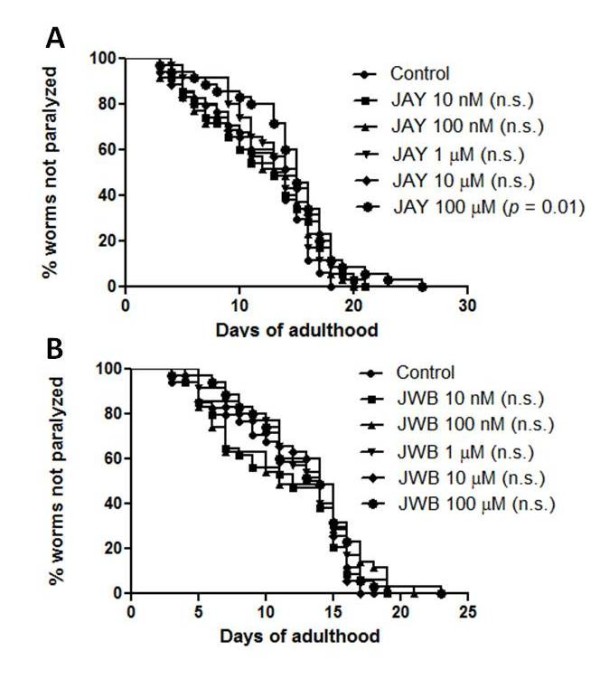
**Aβ-induced paralysis in *C. elegans *strain CL2006 fed with choline analogs, JAY2-22-33 and JWB1-84-1**. (A) Effect of JAY2-22-33 at concentration of 10 nm, 100 nm, 1 μM, 10 μM and 100 μM on Aβ-induced paralysis. (B) Effect of JWB1-84-1 at concentration 10 nm, 100 nm, 1 μM, 10 μM and 100 μM on Aβ-induced paralysis. Synchronized eggs of CL2006 *C. elegans *were maintained at 20°C, on the 35 × 10 mm culture plates (~35 eggs/plate) containing vehicle (control) or compound. The treatment was given to the worm from egg stage onward until the worm completely paralyzed (35 worms in each experiment, 3 independent experiments). n.s. = not significant

The worms were also treated with either JAY2-22-33 or JWB1-84-1 at concentration ranging from 10 nM to 100 μM. JAY2-22-33 at concentration 100 μM significantly delayed Aβ-induced paralysis (*p*= 0.01, Figure 5A). However, none of any concentrations of JWB1-84-1 delay Aβ-induced paralysis (Figure 5B).

### Investigate mechanism of action of JAY2-22-33 using RNAi experiment

**Figure 6:**
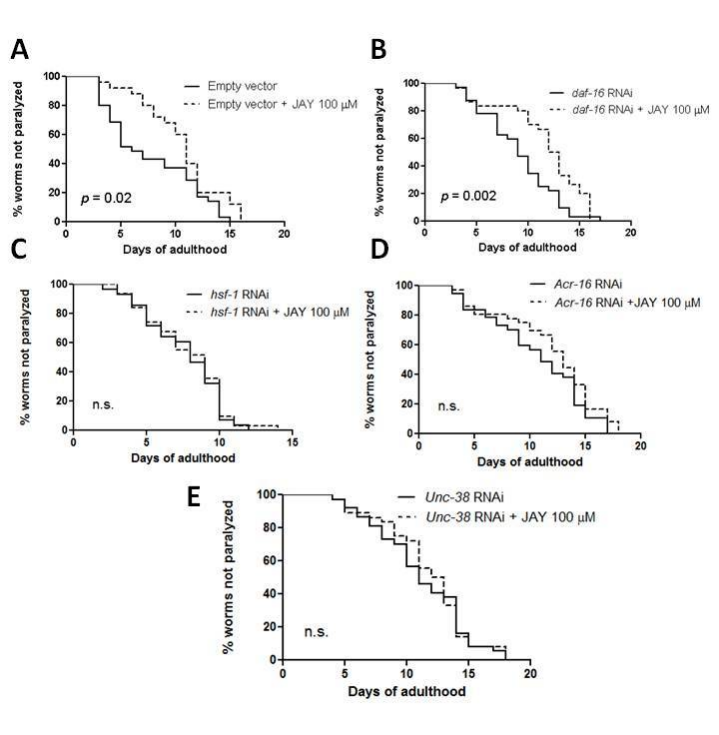
**Paralysis in CL2006 with or without DAF-16, HSF-1, ACR-16 and UNC-38 knock down by RNAi**. (A) Paralysis in CL2006 treated with or without JAY2-22-33 in *C. elegans *fed with vector control RNAi. Solid line, worms grown on bacteria containing empty vector alone; dotted line, worms grown on bacteria containing empty vector and treated with JAY2-22-33. (B) Paralysis in CL2006 treated with or without JAY2-22-33 in *C. elegans *fed with RNAi to down regulate DAF-16. Solid line, worms grown on *daf-16 *RNAi bacteria alone; dotted line, worms grown on *daf-16 *RNAi bacteria and treated with JAY2-22-33. (C) Paralysis in CL2006 treated with or without JAY2-22-33 in *C. elegans *fed with RNAi to down regulate HSF-1. Solid line, worms grown on *hsf-1 *RNAi bacteria alone; dotted line, worms grown on *hsf-1 *RNAi bacteria and treated with JAY2-22-33. (D) Paralysis in CL2006 treated with or without JAY2-22-33 in *C. elegans *fed with RNAi to down regulate ACR-16. Solid line, worms grown on *acr-16 *RNAi bacteria alone; dotted line, worms grown on *acr-16 *RNAi bacteria and treated with JAY2-22-33. (E) Paralysis in CL2006 treated with or without JAY2-22-33 in *C. elegans *fed with RNAi to down regulate UNC-38. Solid line, worms grown on *unc-38 *RNAi bacteria alone; dotted line, worms grown on *unc-38 *RNAi bacteria and treated with JAY2-22-33 (100 worms in each experiment, 3 independent experiments). n.s. = not significant

To identify the role of insulin signaling pathway and nAChRs in mediating the protective effects of JAY2-22-33 against Aβ toxicity, we performed RNAi knock-down of *daf-16*, *hsf-1*, *acr-16*, and *unc-38 *in transgenic *C. elegans *expressing human Aβ. It has been revealed recently that DAF-16, HSF-1 and insulin signaling pathway play a role in the protection against Aβ toxicity [21,24]. It is also well known that *C. elegans *FOXO transcription factor DAF-16 is a key mediator for regulating longevity and stress resistance [25-27]. To test whether DAF-16 and HSF-1 are required for the protective effect of JAY2-22-33 against Aβ toxicity, we performed the experiment by using RNAi knock down of DAF-16 or HSF-1 expression. We found that JAY2-22-33 at concentration 100 μM significantly delayed Aβ-induced paralysis in worms grown on bacteria containing empty vector (*p *= 0.02, Figure 6A) and *daf-16 *RNAi (*p *= 0.002, Figure 6B) but not on *hsf-1 *RNAi bacteria (*p *= 0.69, Figure 6C). This result indicated that reducing the activity of HSF-1 abolished the protective effect of JAY2-22-33, suggesting the requirement of HSF-1 for protective effect of JAY2-22-33. On the other hand, JAY2-22-33 at dose 100 μM still significantly delayed Aβ-induced paralysis in worms fed with *daf-16 *RNAi bacteria, indicating that DAF-16 is not required for the protective effect of JAY2-22-33.

We also found that JAY2-22-33 at a concentration 100 μM did not delay Aβ-induced paralysis in worms fed with bacteria containing either *acr-16 *(*p *= 0.23, Figure 6D) or *unc-38 *RNAi bacteria (*p *= 0.82, Figure 6E), indicating that both nAChRs were required for the protective effect of JAY2-22-33.

In this study, we investigated the effects of JAY2-22-33 and JWB1-84-1 on Aβ levels in an *in vitro *model using a mouse neuroblastoma N2a cell which can express Aβ in an inducible manner [28]. We found that both JAY2-22-33 and JWB1-84-1 significantly reduced Aβ levels in this mutant cell line. This result indicated that these compounds may have the effect on Aβ processing or clearance. In addition, JAY2-22-33 and JWB1-84-1 also protected against Aβ toxicity in rat primary cortical neurons. In a previous study, we found that these compounds showed the potential effect on improving cognitive function in Aβ transgenic mice [14]. The data presented here support the idea that these compounds may have potential benefit in Alzheimer's disease models in addition to their potent cytoprotective effect found *in vitro *[13].

We then evaluated the effect of JAY2-22-33 and JWB1-84-1 in a *C. elegans *model of Aβ toxicity. *C. elegans *is a genetically and functionally well characterized organism that is easy to maintain, can be cultivated in large numbers and it has a relatively short life span, allowing AD-related studies to be performed in a relatively brief period of time. *C. elegans *does not produce endogenous Aβ, thus, providing the opportunity to express and study human Aβ specifically. Human Aβ has been expressed in the body wall muscle cells in *C. elegans *[19]. After the expression and deposit of Aβ in the muscle cells, the worms became progressively paralyzed indicating deterioration in the function of the body wall muscle cells expressing Aβ. We used nicotine as a positive control because of its effectiveness in reducing Aβ levels in previous studies [6,7,29] and its neuroprotective effect against Aβ toxicity in rat primary cortical neuron shown in this study. Our results showed that nicotine reduced Aβ toxicity by significantly delayed Aβ-induced paralysis. However, this beneficial effect of nicotine was only observed with lower concentrations. Higher concentrations of nicotine may be toxic to the worm and in other studies found that high concentrations have been associated with spastic paralysis [30]. Our results are thus in agreement with other study showing that nicotine has benefit effects in various models of AD [6,7,29,31], although one study indicated the absence of effect of nicotine in transgenic mice model of AD [32]. Similar to nicotine, JAY2-22-33 significantly delayed Aβ-induced paralysis indicating its protective effect in this model of Aβ toxicity. However, JWB1-84-1 at concentrations up to 100 μM did not show the protective effect in this model. We further investigated whether the protective effect of JAY2-22-33 is relevant to the reduction of toxic species Aβ oligomer. We found that JAY2-22-33 did not reduce the level of Aβ oligomer suggesting that protective effect of JAY2-22-33 involves other mechanisms (data not shown).

Another advantage of *C. elegans *is that it can be used as a tool to identify the potential targets of active compounds. Despite its phylogenetic differences, *C. elegans *shares a large number of genes and biological pathways with mammalians. About 50-60% of the *C. elegans *genes are homologous to human genes [33]. Additionally, double-stranded RNA interference (RNAi) is useful method for gene disruption in *C. elegans*. So we took these advantages to identify the mechanism of action of JAY2-22-33 on delaying Aβ-induced paralysis. Significant evidence indicates that insulin receptor/IGF-1 receptor signaling plays a role in AD [34] and has direct effect on the metabolism and clearance of Aβ [35,36]. Cohen and coworkers showed that knocking down DAF-2, the homolog of the mammalian IR/IGF-1R, reduced Aβ toxicity [21,24]. DAF-16 and HSF-1, which are down-stream components of insulin signaling pathway, have been shown to play an important role in reducing Aβ toxicity. DAF-16, which is homologous to human FOXO1 [37], regulates the less toxic high molecular aggregation process whereas HSF-1 modulates Aβ disaggregation process. By knocking down these genes using RNAi method, our results indicated that HSF-1 is required for the protective effect of JAY2-22-33 whereas DAF-16 is not required.

The heat shock transcription factor, HSF-1, regulates expression of many different heat-inducible target genes such as heat shock proteins. HSF-1 has been implicated in modulating both longevity [38-40] and proteotoxicity [21,24,38]. Reduction of insulin/IGF-1 signaling protects worms from proteotoxicity associated with the aggregation of Aβ. To alleviate proteotoxicity in worms, insulin/IGF-1 requires HSF-1 to modulate the disaggregation process of Aβ. Our results showed that HSF-1 is required for the protective effect of JAY2-22-33, so it is possible that JAY2-22-33 may mediate the activity of HSF-1 through the insulin signaling pathway. However, further studies will be necessary to clarify how JAY2-22-33 act on this pathway.

Nicotinic acetylcholine receptors (nAChRs) are a family of highly conserved pentameric channels used extensively in both vertebrate and invertebrate neurotransmission. This receptor accounts for a major component of the synaptic response at the *C. elegans *neuromuscular junction [41]. In this study we asked the role of nAChR in mediating protective effects of JAY2-22-33 against Aβ toxicity by using mutant acr16, which is homologous to human α7 nAChR, and unc-38, which encodes α acetylcholine receptor subunit [41-43]. We reasoned that if protection against Aβ toxicity by JAY requires either of the proteins, then the observed delaying of paralysis would be abolished. We found that both ACR-16 and UNC-38 were required for the protective effect of JAY2-22-33. However, the relationship between nAChR and Aβ metabolism in *C. elegans *has not been established yet. In contrast, it has already been shown that the loss of nAChRs enhances Aβ toxicity in a mouse model of AD [9]. Our results in *C. elegans *indicated that the presence of nAChRs were necessary to mediate the protective effect of JAY2-22-33.

Insulin signaling pathway is a key regulator of aging and longevity in worms [44], flies [45], mice [46] and human [47-49]. Since we found that the protective effect of JAY2-22-33 is mediated through the insulin signaling pathway, we further investigated whether JAY2-22-33 prolongs life span. Our result showed that JAY2-22-33 did not extend life span (data not shown)

## Conclusion

In conclusion, this study provided information for decoding potential multitarget mechanism that mediates neuroprotective effect of choline analog, JAY2-22-33. Via the use of *C. elegans *as a model of Aβ toxicity, we determined that JAY2-22-33 significantly delayed Aβ-induced paralysis and that this protective effect required both the insulin signaling pathway and nAChRs. It should be noted that none of the choline analogs we have evaluated to date (including JWB1-84-1 and JAY2-22-33) exhibit a potent ability to displace α_7 _ligands in competition binding assay. JWB1-84-1 and JAY2-22-33 were also recently evaluated in a neurotransmitter screen (ligand displacement assays) at a single 10 μM concentration at 40 additional (potential) drug targets. There were no potent interactions at any of the receptors/ion channels evaluated (as would be indicated by > 90% displacement). These data (not shown) were generously provided by the National Institute of Mental Health's Psychoactive Drug Screening Program, Contract # HHSN-271-2008-00025-C (NIMH PDSP). Thus, the molecular mechanisms of the neuroprotective effects of JWB1-84-1 and JAY2-22-33 are not fully resolved at this time. Potential effects at allosteric receptor sites and other signaling pathways will be the focus of future experiments.

## Methods

### JWB1-84-1 and JAY2-22-33 synthesis

The detail for synthesis of N-(2-hydroxyethyl-N'-(3-pyridylmethyl)-piperazine JWB1-84-1 and 2(N-methyl, N-methyl-3-pyridoamino) ethan-1-ol (oxalate salt) JAY2-22-33 was previously described [50].

### Cell culture

#### N2a cells (Aβ expressing mutated cell line)

The N2a cell line expresses a Swedish mutation in APP 695 and another mutation in PS1 whereby exon-9 is deleted. These double mutations are similar to the ones seen in early stage familial Alzheimer's disease [51]. N2a cells produce high levels of Aβ upon stimulation with sodium butyrate. This cell line was a gift from Dr. H. Xu at University of California in San Diego, CA, USA. This cell line is used to test the effect of JWB1-84-1 and JAY2-22-33 on the levels of amyloid species extracellularly (in the medium)

### Primary cortical neurons

Cortical neurons were harvested from rat pups (postnatal day 0) and plated at the density of 100,000 cells per well in 96 well plates. The cells were maintained in neurobasal media with supplement of B27, 1% pen/strep and 0.5 mM Glutamine. The cell cultures were incubated in a 5% CO_2_, humidified incubator at 37°C. The media was changed every three days.

### Quantitation of Aβ using ELISA

Ninety six-well plates were coated with 100 μl 6E10 (capture antibody) 20 μl/10 ml in PBS overnight with rocking at 4°C. The plates were then washed with PBST (0.05% Tween 20) 5 times. Blocking buffer (PBS with 1% BSA and 5% horse serum) 200 μl was added and incubated 2-4 hr at room temperature. Increasing concentration of the conditioned medium or Aβ standard 50 μl was added to the plates and incubated overnight at 4°C. After 3 washes, 100 μl of 4G8 biotinylated antibody (5 μl/10 ml in PBS containing 1% w/v BSA) was added and incubated for 2 hr at room temperature. After washing 5 times with PBST, 100 μl of Streptavidin-horseradish peroxidase (1:200 dilution in PBS with 1% BSA) was added and incubated for 30 minutes at room temperature. One hundred microlitre of tetramethylbenzidine (TMB) which is a substrate for HRP was added to the well and incubated at room temperature for 15 min. Stop solution (2 N H_2_SO_4 _or 1 M H_3_PO_4_) 50 μl was added and the absorbance of the well was read at 450 nm.

### Cell viability assay

This assay was performed using Invitrogen Vybrant^® ^MTT Cell Proliferation Assay Kit (Invitrogen V-13154). The cortical neurons were cultured for 7 days before the experiment. On the day of experiment, the primary cortical neurons were incubated with vehicle or with various concentrations of compound Nicotine, JWB1-84-1 or JAY2-22-23 for 24 hours. The cells were washed and challenged with 100 μM Aβ for 24 hours. Cells were then washed with fresh Neurobasal media and 100 μl of fresh Neurobasal media plus 10 μl of 12 mM MTT stock solution were added to each well. Cells were incubated at 37°C for 4 hours. 100 μl of the SDS-HCl solution were added to each well and mixed thoroughly and incubated for another 4 hours. The absorbance was measured at 570 nm.

### Drug treatment (in *C. elegans*)

Nicotine (Nicotine hydrogen tartrate salt; Sigma-Aldrich, USA), JWB1-84-1 (N-(2-hydroxyethyl-N'-(3-pyridylmethyl)-piperazine HCl) and JAY2-22-33 (2 (N-methyl, N-methyl-3-pyridoamino) ethan-1-ol (oxalate salt)) were added to the OP50 bacteria to a desired final concentration. The treatment was given to the transgenic worm from egg stage onward.

### *C. elegans *strains

The wild type *C. elegans *strain N2, the transgenic strain CL2006 were obtained from the Caenorhabditis Genetic Center (University of Minnesota). The construction and characterization of transgenic *C. elegans *strain CL2006 has been described previously [52]. Maintenance of all strains was routinely performed at 20°C on Nematode Growth Medium (NGM) plates with *Escherichia coli *strain OP50 as a food source as previously described.

### Paralysis assay

The reproductive adults of transgenic *C. elegans *strain CL2006 maintained at 20°C were transferred to the 35 × 10 mm culture plates containing either a vehicle or drug, and allowed to lay eggs for 4-6 h, producing age-synchronized groups. The worms were tested everyday for paralysis. To identify the paralysis, each worm was gently touched with a platinum loop. The worms were scored as paralyzed when they displayed no body movement when prodded with a platinum loop.

### RNA interference (RNAi)

RNAi was performed in *C. elegans *by feeding the worms with dsRNA-containing bacteria. *C. elegans *was fed with *E. coli *HT115 strains expressing dsRNA specific to *daf-16*, *hsf-1*, *acr-16 *and *unc-38 *gene. After 3-4 h, worms were removed and eggs were permitted to mature to L4 young larvae. These worms were considered as the first generation (F1). Then, the L4 larvae (F1) were transferred to another plate containing dsRNA and allowed to lay eggs. The resultant adult worms were considered as the second generation (F2) and were used for the paralysis assay.

### Data analysis

Each of the data points depicted in the figures represents the mean ± S.E.M. Significant differences between groups were assessed by one-way analysis of variance with statistically significant differences accepted at the *p *< 0.05 level. Post hoc analysis was performed using Dunnett's method. GraphPad Prism 5 software was used for the paralysis and survival analysis. *p *value calculations were made between treated and untreated animals using the log-rank (Mantel-Cox) test.

## List of abbreviations

Aβ: β-amyloid; *C. elegans*: *Caenorhabditis elegans*; nAChRs: nicotinic acetylcholine receptors; AD: Alzheimer's disease; ACh: acetylcholine; AChE: acetylcholinesterase; AChEIs: acetylcholinesterase inhibitors; IGF-1: insulin-like growth factor-1; HSF-1: heat shock factor-1; NMDA: N-methyl-D-aspartate; RNAi: RNA interference; APP: amyloid precursor protein; PS 1: presenilin 1; PBS: phosphate buffered saline; PBST: phosphate buffered saline tween 20; BSA: bovCalibri is acceptable, can you checkline serum albumin; TMB: tetramethylbenzidine; *Escherichia coli*: *E. coli*; dsRNA: double-stranded RNA; SEM: Standard error of mean.

## Competing interests

The authors declare that they have no competing interests.

## Authors' contributions

RK carried out all drug treatments, behavioral assays in C. elegans and statistical analysis, and drafted the manuscript. MA carried out the N2a cell treatment, quantitative Aβ assay and data analysis. BA and WB performed drug treatment of PC12 cells, cell viability assay and data analysis. AT and JB provided the compounds they developed, and participated in the design of the study and manuscript revision. YL coordinated the study design and manuscript submission. All authors read and approved the final manuscript.
